# Bestrahlen oder nicht bestrahlen bei älteren Patientinnen mit Low-Risk-Mammakarzinom? Erkenntnisse aus den 10-Jahres-Daten der PRIME-II-Studie

**DOI:** 10.1007/s00066-023-02071-z

**Published:** 2023-03-27

**Authors:** Elena Sperk

**Affiliations:** Mannheim Cancer Center, Universitätsmedizin Mannheim, Medizinische Fakultät Mannheim, Universität Heidelberg, Theodor-Kutzer-Ufer 1–3, 68167 Mannheim, Deutschland

## Hintergrund

Die randomisierte Studie PRIME II wurde aufgesetzt um Level-I-Evidenz zum Verzicht der Radiotherapie (RT) bei älteren Patientinnen mit einem frühen Mammakarzinom und positiven Hormonrezeptoren zu generieren.

## Patientinnen und Methoden

Einschlussfaktoren waren: Alter ≥ 65 Jahre, positive Hormonrezeptoren, cN0 und T1/2 bis 3 cm. Alle Patientinnen erhielten eine brusterhaltende (BET) und endokrine Therapie. Randomisiert wurde zwischen dem Verzicht auf eine adjuvante RT und der adjuvanten Ganzbrustbestrahlung (WBRT) mit 40–50 Gy. Der primäre Endpunkt war die Lokalrezidivrate (nach 5 Jahren). Zusätzlich wurden noch regionale Rezidive (Lymphabfluss), das brustkrebsspezifische Überleben, die Metastasierungsrate und das Gesamtüberleben untersucht.

## Ergebnisse

Es wurden 1326 Patientinnen mit einem medianen Follow-up von 9 Jahren eingeschlossen (*n* = 658 mit RT, *n* = 668 ohne RT). Die kumulative Lokalrezidivrate nach 10 Jahren lag bei 9,5 % in der Gruppe ohne RT und bei 0,9 % mit RT (Hazard Ratio [HR] 10,4; *p* < 0,001). Die sekundären Endpunkte zeigten sich nicht signifikant unterschiedlich.

## Schlussfolgerungen der Autoren

Die Autoren kommen zu dem Schluss, dass der Verzicht auf Radiotherapie in diesem Kollektiv trotz erhöhtem Lokalrezidivrisiko vertretbar (da < 10 %) und vor allem bei hochpositiven Hormonrezeptoren und endokriner Therapie über 5 Jahre auch sicher sei.

## Kommentar

Die PRIME-II-10-Jahres-Daten waren lang erwartet. Überraschend ist allerdings die Schlussfolgerung der Autoren! Artikel wie „Radiotherapy: no benefits for older patients with early breast cancer“ [1] und die Botschaft der Autoren in diversen (sozialen) Medien verzerren die eigentlichen Ergebnisse maximal! Aus der Perspektive einer Radioonkologin erscheint eine hochsignifikante Reduktion der Lokalrezidivrate (HR 10,4!) durch eine Radiotherapie mehr als eindeutig für dieses multimodale Konzept (Operation, endokrine Therapie, Radiatio) zu sprechen.

Wie kommen die Autoren also auf die o. g. Headline? Interessant ist vielleicht die Tatsache, dass Ian Kunkler (Initiator und Erstautor der Studie) klinischer Onkologe ist. Dass die medikamentöse Therapie Onkologen näher und der Fokus damit nicht auf der lokalen Kontrolle liegt, ist nachvollziehbar und schlägt sich auch in den meisten onkologischen Studien in Form der primären Endpunkte nieder. In dieser Studie wurde allerdings der Fokus auf das Lokalrezidiv gelegt, denn es ist bereits bekannt, dass die RT in diesem Kollektiv keinen Effekt auf das Überleben hat. Somit ist der gewählte primäre Endpunkt in PRIME II (Lokalrezidivrate) auch folgerichtig der eigentlich interessante Punkt! Dieser fällt aber am Ende zugunsten des sekundären Endpunkts (Gesamtüberleben) weg, obwohl die Autoren im Methodenteil selbst schreiben, dass die sekundären Endpunkte nicht den Ansprüchen einer Hypothesentestung genügen (z. B. keine Adjustierung auf multiple Testung). Am Ende scheinen selbige dies aber nicht so genau zu nehmen. Nichtsdestotrotz lassen sich sehr wichtige Erkenntnisse aus der Studie ziehen!

### Wichtigste Erkenntnisse aus PRIME II

Die sehr gute lokale Kontrolle nach RT bei älteren Patientinnen mit Low-Risk-Mammakarzinom bestätigt ergänzend zu den bekannten Daten aus z. B. CALGB 9343 [2], dass die RT hoch wirksam ist (Senkung der Lokalrezidivrate um den Faktor 10,4).

- Die Autoren versuchen, den Verzicht auf RT mit den möglichen Toxizitäten (z. B. Kardiotoxizität) der Radiatio zu untermauern, ohne selbst diese Daten erhoben zu haben. Hier kann allerdings das gleiche Argument vonseiten der Radioonkologie herangezogen werden wie von den Autoren selbst verwendet: Die RT hat in dieser Studie zwar keinen positiven, aber auch keinen negativen Einfluss auf das Gesamtüberleben und ist somit nicht besonders toxisch!
- Die Autoren sprechen Alternativen wie Teilbrust-RT und neuere Bestrahlungstechniken in zwei Sätzen an und verweisen auf vermeintliche Schwierigkeiten (Zielvolumendefinition) – ohne Literaturhinweis. Sie erwähnen nicht, dass es mittlerweile für viele Teilbrust-RT-Techniken sehr gute Daten gibt, die zeigen, dass die Zielvolumendefinition funktioniert, ob perkutan [3], mittels Brachytherapie [4] oder mit intraoperativen Techniken (IORT; [5]). Ebenso verlieren die Autoren kein Wort über moderne Techniken mit z. B. Atemanhalt, „gating“, „image guidance“ oder sonstige Fortschritte, die Toxizitäten minimieren können.
- PRIME II untermauert schließlich die S3-Leitlinien-Empfehlung [6]: In einer sehr günstigen Low-Risk-Konstellation mit entsprechend geminderter Lebenserwartung kann ein Verzicht unter Inkaufnahme des erhöhten Risikos für ein Lokalrezidiv erwogen werden. Denn auch in dieser Analyse wird deutlich: Die Gruppe ohne RT erreicht kein Plateau bei den Lokalrezidiven. Stattdessen ist das Risiko auch nach vielen Jahren immer noch gegeben und setzt sich immer weiter fort. Warum sollten wir also Patientinnen mit einer Lebenserwartung von > 10 und teilweise noch > 20 Jahren eine hoch wirksame Therapie vorenthalten? Überraschend ist in diesem Zusammenhang auch, dass die Auswirkungen eines Lokalrezidivs in der Diskussion weitestgehend außer Acht gelassen werden: finanzielle Auswirkungen auf das Gesundheitssystem durch erneute Therapien auf der einen Seite oder körperliche, psychische und finanzielle Belastung der Patientinnen und damit die vermutlich negativen Auswirkungen auf die Lebensqualität auf der anderen Seite.

## Fazit

Die Radiatio ist mit diesen Spitzenergebnissen eine wichtige Säule im multimodalen Therapiekonzept des Mammakarzinoms – auch bei älteren Damen mit günstiger Tumorbiologie und endokriner Therapie. Es scheint in Anbetracht der partizipativen Entscheidungsfindung und des demografischen Wandels fast schon unethisch den Patientinnen gegenüber zu sein, ihnen eine solche hocheffektive Therapie kategorisch vorzuenthalten. Ergänzend dazu gibt es mittlerweile viele alternative RT-Möglichkeiten, um individuelle und nebenwirkungsarme Therapiekonzepte zu gestalten.

*Elena Sperk, Mannheim*
